# Medicine and psychiatry in Western culture: Ancient Greek myths and modern prejudices

**DOI:** 10.1186/1744-859X-8-21

**Published:** 2009-10-07

**Authors:** Michele Fornaro, Nicoletta Clementi, Pantaleo Fornaro

**Affiliations:** 1 Dipartimento di Psichiatria, Università di Genova, Genoa, Italy; 2 Psychopharmacology Unit, Bristol University, UK

## Abstract

The origins of Western culture extensively relate to Ancient Greek culture. While many ancient cultures have contributed to our current knowledge about medicine and the origins of psychiatry, the Ancient Greeks were among the best observers of feelings and moods patients expressed towards medicine and toward what today is referred to as 'psychopathology'. Myths and religious references were used to explain what was otherwise impossible to understand or be easily communicated. Most ancient myths focus on ambiguous feelings patients may have had towards drugs, especially psychotropic ones. Interestingly, such prejudices are common even today.

Recalling ancient findings and descriptions made using myths could represent a valuable knowledge base for modern physicians, especially for psychiatrists and their patients, with the aim of better understanding each other and therefore achieving a better clinical outcome. This paper explores many human aspects and feelings towards doctors and their cures, referring to ancient myths and focusing on the perception of mental illness.

## Background

The origins of Western culture are extensively related to Ancient Greek culture, and many current aspects of human behaviour and social organisation still rely on these roots. This observation, having relevant implications for the whole of medical practice and therefore for psychiatry too, is hardly ever recalled, even among well educated people (as physicians are expected to be). As a consequence, many aspects of human life, such as fear, pain, convictions, expectations and prejudices against medicine (especially the ones against psychiatry and its practitioners), illness and death today seem quite disregarded. Nevertheless, such human aspects, already extensively investigated many centuries ago by different cultures, are often applied to mythology, leading to understandings still relevant in modern times.

The honourable, yet too often disregarded, Confucius's (551 to 479 BCE) quote 'study the past if you would divine the future' perfectly applies to the above-mentioned scenario, reminding us that concepts and observations from different ages and cultures may be equally true even among different cultures of heterogeneous origins and chronological settings.

From a medical and clinical psychiatric perspective, Ancient Greek culture and myths represent a valuable prototype for greater comprehension of our modern practice, with the aim of instigating better clinical compliance, treatment adherence and outcomes.

## Stigmatisation, myths and prejudices against mental illness

Prejudice and discrimination against mental illness have been reported since ancient times, while both somatic and non-somatic illnesses were traditionally considered as a sort of 'punishment' for a guilty patient. The Ancient Greek word 'aítia' ('guilty', 'responsibility'), also links with the beginning of the word 'aetiology' and other medically related words as well. Prejudices against mental illness had further developed by the period from the Middle Ages to the 17th century, when the French physician Perdulcis (1545 to 1611) first introduced psychiatric nosology describing clinical pictures as 'demonopatia mania' and 'demonic possession' provoked by the blending of 'evil spirit' with 'Hippocratic humors' [1].

An example of such an approach to mental illness is the one provided by hysteria, whose somatic anaesthesia, in the sense of pain relief, was considered to be due to demonic interventions until the late 17th century, before being though of as an illness, yet it is still seen today in a detrimental way, since seldom identified as a factitious disorder [2].

Today, magical interpretations, myths and prejudices against mental illness are not uncommon, often leading to clinical worsening of the patient's conditions with resulting loss of self-esteem, fear, social retirement and acting out among other issues.

High failure rates in early recognition of depression have been reported among general practitioners, possibly due to a prevalence of somatic presentation at onset. Indeed, somatic symptoms are 'easier' and quicker to be diagnosed and to be 'accepted', and this is probably why many patients (usually 'unconsciously') exhibit such manifestations instead of non-somatic, 'masked', ones.

A 'masked depression', defined as one with almost exclusively somatic presentations, is often reported among specific populations or social conditions as an expression of the 'pathoplastic' effect of culture [3]. As a consequence, delay in adequate treatment can often provoke further impairment and lead to a poorer outcome.

Stigma and prejudice against mental illness, though still far from being a thing of the past, has been progressively addressed in the media and by various organisations. For example, the National Alliance on Mental Illness (NAMI) counts more than 185,000 members in USA involved in spreading greater knowledge of the clinical and social phenomenon of mental illness. NAMI also cooperates with the American Psychiatric Association (APA) in pursuing the goal of introducing acts overcoming social and employment laws that are discriminatory to the mental ill.

Similar objectives are the ones the World Health Organization (WHO) is pursuing along with many others societies and organisations among different countries. In the UK, the 'Changing Minds Stigma Campaign' introduced in 1997 by the Royal College of Psychiatrists had the aim of spreading knowledge about the phenomenon of mental illness among the general population, while the World Psychiatric Association (WPA) mainly focuses on reducing stigma against schizophrenia.

The above-mentioned organisations and societies, and many others besides, also focus on reducing stigma against psychotropic drugs, as they usually represent the core therapy to treat psychiatric conditions. In fact, most discrimination and fear related to psychiatric drugs is due to the potential side effects common to first generation treatments.

For example, typical antipsychotics, an effective and valuable class of drugs, have been repeatedly reported to potentially induce extrapyramidal syndrome (EPS), tardive dyskinaesia (TD) and other side effects [4], while newly introduced, safer yet still effective atypical antipsychotics are still not as 'popular' in the media or as well known by patients [5], especially in Europe and Australia compared to USA.

Further complicating the perception patients and the general population have of psychiatric disorders is the fact that in most cases psychiatric conditions can manifest with heterogeneous clinical pictures during the lifespan of the patient [6].

Sleep disturbances, motor retardation or agitation and cognition impairments, as many other symptoms, could precede a 'full threshold' disorder or represent a residual one, possibly due to a partial remission and/or an abrupt interruption of pharmacotherapy, leading to a poorer quality of life and further increasing social and interpersonal problems [7].

## Mythology, medicine or both?

Elephants and pigs have long been observed to enjoy the effects of alcohol obtained by eating fallen mangoes or apples that are fermenting. It seems like our ancestors observed and copied the behaviour of such animals and then, being human and having the ability to think and create, developed methods to ensure a continuous alcohol supply. One assumes that the survival value of such a process was then, as now is, the desire for temporary escape from the human condition.

Natural psychotropic substances obtained from plants including tobacco, cannabis, opium, coffee and others provided the needed escape, constituting the so-called 'pre-alcoholic era'.

Both recreational and therapeutic uses for natural resources were pursued at the same time. Looking to their close environment, human beings did not distinguish between substances aimed at treating illness or alleviating pain. Indeed, both purposes were originally considered as indivisible needs and only later were they considered separately. Moreover, somatic and psychic illnesses were initially seen as part of the same whole, therefore being treated using the same substances as well.

Psychic and somatic symptoms were considered as separated phenomena only in later times, before finally being progressively considered once more as different manifestations affecting the 'same body' in the course of the 'same disease'.

Rather than looking for remedies from environmental sources, the human need to search for a cure for pain, illness and death has been addressed by looking to the supernatural and magic. Unsurprisingly, the word 'remedy' derives from the Latin verb 'mederi', which resembles the Latin origin of the word 'medicine' as well. In fact, the main goal of medicine is to provide a cure for pain and illness, independently of the source.

Ancient Greek medicine was a complex practice perceived as something between myth and reality, as an expression of a magical divinatory, hieratic and empirical technical practice. Consequently, ancient medicine is tightly linked with ancient mythology.

An example of such overlap is the one provided by the myth of Asclepius, considered, quoting Pindar (522 to 443 BCE), to be 'the god of medicine' by Ancient Greeks [8].

According to the myth, Asclepius, son of the god Apollo and the nymph Coronis, was born from by the dead body of his mother, an unfaithful wife executed by the goddess Artemis, twin sister of Apollo. This has been considered as the first Caesarean birth delivered from a dead mother. Asclepius was raised by Chiron, a centaur, again an overlap between myth and medical knowledge, considered as the master of medical practice and herbal medicines. Soon the pupil surpassed his master, becoming the 'god of medicine'.

Homer (circa 8th century BCE) also reports Asclepius as being the first to distinguish between medicine and surgery: he gave the power of recovery to his son Podalirius and the ability to treat wounds to Machaon; interestingly, no separation between psychic and somatic conditions was made [9].

Most ancient myths were routinely related to everyday life events. Over 200 temples to Asclepius's were built within the Hellades, representing the first known hospitals. The patients were allowed to rest and to sleep close to the arcades where prophetic dreams took place, probably induced by the unwitting consumption of drugs. During such dreams, they believed they could feel the presence of Asclepius providing them with therapeutic tips and the ability to recovery. In fact, the wizard doctors, dressed up as gods, were the ones giving poisons to the patients at night.

Temples had long been considered the preferred setting for magical and medical rituals, and animals were a frequent presence during such ceremonies. The mythological character of Coronis, the mother of the god of medicine, is also related to the therapeutic ritual performed at the sacred temple of Athena. The name Coronis is derived from the Ancient Greek word 'corònos' meaning 'crows', considered as being related to an ancient diagnostic and therapeutic ritual performed to divine the future. During this practice it was possible to attend to the reincarnation of heroes as ravens or snakes by the intervention of the goddess Athena, also known as the 'mistletoe user'.

Harvesting mistletoe growing over oak branches retained the symbolic meaning of castrating the host tree because the juice from mistletoe berries, seen as the 'oak sperm' (mistletoe was called 'viscum album' in Latin, maybe due to its sperm-like features) was considered to be charged with regenerative powers and therapeutic properties [10]. The word 'mistletoe' is related to the Latin name of the god of medicine, Asclepius, as well, meaning 'what is hanging from the edible oak', or 'esculent', that is to say 'good to be eaten', as mistletoe also means.

It is curious to note that among different civilizations and in different ages, the same substances were used with similar purposes while today most of these significances are all but forgotten. For example, the Celts considered mistletoe sacred too, using it in many religious rituals. This plant was also widely used as a remedy during the Middle Ages and the Renaissance, while by the second half of the 19th into the first decades of the 20th century it was being prescribed for its antihypertensive properties. Today, it just has a symbolic value at Christmas.

Mistletoe is just one among many different herbs and trees used in ancient medical practice; the bark of willow trees surrounding the temple of Athena contained salicylate, an antipyretic and anti-inflammatory drug still widely prescribed by modern physicians (today known to be able to interact with reactive proinflammatory C-protein).

Independently of the myths used to explain medical illnesses and their management, overlaps between legend, religion and medicine had long been widespread among Ancient Greeks until Hippocrates (460 to 377 BCE) first distinguished such entities.

Hippocrates introduced a more modern-like model of depression. He applied the concepts of his essential 'theory of four humors and temperaments' to melancholia ('mélaina' means 'black' while 'cholé' is 'bile'), described as a consequence of an imbalance of the four essential fluids influencing both physical and psychic manifestations. These fluids ('humors') were blood, phlegm, black bile and yellow bile.

As for psychic disorders, the fluid theory was equally valid for somatic manifestations too. According to Hippocratic theory, an imbalance in fluid proportions was considered responsible for a peculiar temperament, possibly leading to severe depression, while a healthy subject, the 'good humor' man, has the four humors in appropriate proportions. Depression and other psychiatric conditions were therefore considered organic disorders.

Modern doctors know that in the course of pancreatic cancer most patients experience severe 'melancholic' depression, almost indistinguishable from primary depression; ancient doctors had already noted this clinical phenomenon, and this is probably why the pancreas was considered the inner site of black bile.

Using the above-mentioned theory, Hippocrates was also the first to describe possible seasonal courses of 'mood' or 'humor' disorders [11]. Most Hippocratic observations represented innovative progresses in the medical field. Yet, they were generally disregarded or 'misunderstood' during the ages that followed, when myth (or religion) influenced the medical and scientific approach.

## Poisons and remedies

'Doctors are men who prescribe medicines of which they know little, to cure diseases of which they know less, in human beings of whom they know nothing' (Voltaire, 1694 to 1778).

Most clinicians know from their everyday practice that a great number of patients show mistrust towards suggested drugs, especially for treatments prescribed to them for the first time and/or by physicians they have not met previously or do not know well or trust. This is probably due to a lack of knowledge and seems to be particularly true in modern psychiatry, which bases most of its therapeutic interventions on the pharmacologic approach.

Patients could perceive drugs aimed at treating their 'inner psychic pain' as 'mysterious' and/or 'unnatural' things. While today the impact of somatic pain and disease is accepted almost everywhere, this is not always true for psychiatric conditions, although possibly manifesting with somatic symptoms as well [12]. In other words, the physical pain reported by some psychiatric patients should be more often go disregarded compared the one reported by "non psychiatric patients"; in many cultures, this may have contributed the somatic expression of psychiatric symptomatology to be a "more acceptable" way to communicate the experienced pain.

Again, looking back to Ancient Greek mythology, it is easy to find many explanations for this kind of mentality, prejudice and concern against the 'doctor and his drugs', which in most cases is still far from being completely overcome even today.

The etymology of the word 'pharmacology' has different meanings. The Ancient Greek word 'phàrmakon' means 'poison' or 'drug', but also means 'lucky charm'. In fact, the 'drug', as a 'poison', is a substance that could lead to the patient's death or, as with a magic 'amulet', it could 'magically' lead to their recovery.

The ambivalence patients experienced towards doctors and their cures has been described in many Ancient Greek myths. One famous Greek myth is that of the Zeus oracle Trophonios, situated in a cavern in the Lebadeia village in the region of Boiotia.

According to the myth, Trophonios was also a sort of 'wizard doctor', able to change the given temperament of a subject to one of an opposite polarity. Patients, most likely catatonic depressed ones, were taken into his presence with the aim of making them recover from severe illness [9]. Curiously, the Novartis trade name for the tricyclic antidepressant molecule imipramine is 'Tofranil', referring to a hoped immediate improvement in the mood of depressed patients.

Most Ancient Greek myths were also adopted by later civilizations. Ancient Roman coins represented Janus Bifrons as a frightening ugly character with two bearded faces looking in opposite directions (a double-headed character had already appeared on Greek coins in Amphipolis and Thessalonica) [9]. The 'double-face' profile of the character and the two opposite possible outcomes of the 'therapy', perfectly resemble the concept of ambivalence toward medicine and its practitioners.

One of the most curious aspects of Ancient Greek mythology is that in most cases the same myth, based on a relatively simple structure, is used to 'explain' different (often complex) concepts not elsewhere understood. This applies to complicated concepts and life events experienced by the patient, such as pain, illness and death.

Again, referring to the myth of Asclepius, it includes elements closely associated with the magical and irrational aspects of medical practice and its remedies. The expectation of recovery coexists with the worry the same therapeutic intervention might also be harmful. Ancient Greeks believed Asclepius received from Athena two vials of blood she obtained from the body of the Gorgon Medusa. This blood is a perfect example of the concept of 'phàrmacon'. If obtained from the left side of the Gorgon Medusa's body, it is a deadly poison, while if taken from the right side, and managed by Asclepius in person, it has the property to bring back life to a dead body; another interesting concept being that the same drug could be a poison or a remedy depending on who administers it. The role played by the 'iatròs' or 'iatèr' or 'doctor healer', is also described by Homer as 'equal to the gods' and as an 'extraordinary heroic' one.

These kinds of symbolism undoubtedly influenced following cultures too. For example, in Christian iconography the physician is sometimes identified with the saviour, as portrayed in early 17th century oil painting 'Christ the Physician' (Werner Van Den Valckert, 1583 to 1627), representing Christ performing the urine test [13].

## Emotions and beliefs patients have toward medicine and doctors

The emotions, beliefs, attitudes and behaviours described in Greek mythology refer to feelings, moods and expectations that are often reported today by psychiatric patients.

Regression, distress, fear, expectations and hopes resulting from pain and sufferance may all affect and alter the perception patients have about their illnesses, their doctors, and their medical treatment. Often their identities, and therefore their clinical condition, reveal supernatural/irrational aspects that the psychiatrist needs to consider.

These aspects become more evident as the nature and/or the cause of the illness to the patient (and to the doctor too) is more obscure. The patient could also feel his condition as more threatening and stressful depending on the communication style used by the doctor. Finally, the psychological setting the psychiatrist works in is different from the one of other physicians, as therapeutic instruments may vary.

The therapeutic effects, as well as the side effects of psychiatric drugs, result from a large number of heterogeneous factors; they could also be related to the patient's characteristics such as age, sex, physical condition, culture and personality. Another important observation is the fact that psychiatric medications are often perceived as different from general medical ones.

Curiously, the same substances can be used for different purposes: for example the metoclopramide molecule was first introduced as an antipsychotic medication and it is now prevalently used as a procinetic drug (dopamine receptors are present both in the central nervous system (CNS) and the peripheral nervous system (PNS)).

Indeed the patient could have different feelings toward the same therapy depending on the purpose it is given for and depending on the specialty of the prescribing doctor. The individual personality traits of the psychiatrist, their training, experience and culture could strongly influence the 'doctor-patient relationship', possibly affecting the therapeutic compliance and outcome.

The main concern of physicians focuses on the need to overcome recurrent attitudes of distrust towards prescribed drugs, especially psychiatric ones. In order to make this possible, psychiatrists should try to make the pill more desirable for the patient.

The painting of saints Cosmas and Damian by Burgos (circa 1495) at the Welcome Institute in London represents the two characters as caregivers providing pills. There are two kinds of pills: the red and bitter and the golden and sweet ones; the latter ones are a result of a gilding process, again a 'magical' representation of the medical practice.

Today it is still a common saying to 'take the bitter pill' or 'take the gilded pill', meaning that the way the drug it is introduced to the patient could have a significant impact on the patient's feelings toward it, and that an accurate preventive explanation on possible (side) effects by the doctor could further increase the compliance [14].

## Placebo, nocebo effects and polypharmacy

Patients often show high expectations about recovery, rapidly followed by bitter disappointment and sometimes by 'interpretative' reactions: ' [...] I fell ill as the doctor gave me the wrong medicine...' or '[...] I had magnetic resonance imaging, and something went wrong in my brain'. Most of these expectations play a significant role in compliance and outcome.

The concept of 'placebo' refers to the efficacy of an inert biological substance (usually, 'a sugar pill' or any dummy medication) with any therapeutic activity; the patient taking it is unaware of its true composition, assuming they are being given a regular drug [15].

Interestingly, the placebo effect has been 'described' since ancient times: Homer suggested that the same drug could be more effective depending on the caregiver, and if the patient considers the therapist to be an authoritative person or gifted with specific abilities, the outcome may improve. Homer reported that wounded heroes would have a better outcome if they were treated by Helen of Troy in person [9].

The 'first reported placebo effect' was therefore probably documented by the Ancient Greeks. While the placebo concept is quite popular among modern medical practitioners, the 'nocebo' one is not so well known. This latter concept refers to the 'quality inherent in the patient, not the remedy' [16].

As a patient could have 'good' expectations with regard to a treatment, they could also have 'bad' ones. These clinical observations have been investigated by neuroimaging studies. Studies on the placebo effect using positron emission tomography (PET) techniques and pain stimulation reported an activation of endogenous opioid-mediated transmission at the anterior cyngulate cortex, orbitofrontal cortex and insular lobe, amygdala, nucleus accumbens, periacqueductal grey matter and an activation of dopaminergic transmission at the ventral basal ganglia and nucleus accumbens.

Dopaminergic activity and hopioid transmission at the nucleus accumbens has also been reported to be directly related to the placebo response rate. Neuroimaging studies on nocebo effects have focused on the activation of dopaminergic endorphinergic transmissions [17].

Beside placebo and nocebo effects, another important concept is that of 'polypharmacy'. As stated by Hollister, 'the combinations of psychopharms are used far more often than experimental evidence or common sense dictates. Often awkward combinations of drugs arise because no one has taken time to evaluate the changing goals of treatment for a patient, but has simply added new drugs to old treatment with psychotherapeutic drugs requires thought, not reflexes' [18].

Often 'unconscious' elements may also heavily bear on the treatment relationship and outcome. For instance, a patient giving into this dependence shouldn't discontinue their medication, possibly developing symptom clusters inducing polypharmacy [18].

Alternatively, a patient having this dependence may express his anger against the clinician, convinced that whatever the doctor prescribes, it will never be sufficient; again, this could further induce polypharmacy. Considering the above-mentioned considerations, clinicians should have a curious, attentive and open-minded approach to medications, treatments and the 'concept of illness'.

As wizard doctors were supposed to do, modern physicians can heal but also harm or even kill the patient. Ambivalent feelings patients manifest toward the doctor could also lead them to think that the more the effective the treatment, the more the drug could be dangerous (or even life threatening) due to possible side effects.

## 'Ancient psychopharmacology'

Among different ages and cultures, various substances have been proposed as remedies. To mention a few, alcohol, opium, rauwolfia serpentine (dried roots containing reserpine, an alkaloid with antipsychotic and antihypertensive properties) and others have been popular remedies for centuries.

Among others, the Hippocratic humoral theory was the most lasting and influencial one for many centuries. In fact, it influenced the way the diseases and their determining causes were seen by physicians, thus influencing their treatment approaches as well. For example, 'black bile' impairments were still being 'diagnosed' during the Middle Ages and treated with hellebore, or melampodium (literally, 'with black roots'), which probably represents one of the first known 'ex adjuvantibus' therapies.

In fact, hellebor induced massive foul-smelling black loose stools (considered by ancients as black bile) responsible for melancholia. The patient's relatives could admire the extraordinary effects of the cure, but most of the treated subjects were not that happy: patients receiving hellebor often became emaciated and the more unlucky ones died from massive 'melena' (tarry stool) due to sudden gastric haemorrhages and intestinal bleeding. Use of the toxic herb hellebor was continued until late Middle Age, with the name of 'Christmas rose'.

Many legends about hellebor spread through different places during the Middle Ages. Among them, the most popular is probably the one about the shepherd Melampus, whose lambs grazed in luxuriant meadows with laxative effects. By observing their feeding, he decided to experiment with the herb in human beings; this has been considered as among the first reported 'animal to human' medical 'experimentations'. According to the legend, he had a great success, treating the daughters of Proetus, king of Argos (they were convinced to have reincarnated in heifers) for madness. Melampus suddenly became famous among aristocrats as rich people were worried about their constipation. He received a courtesy title of 'purgative doctor', obtaining the wedding ring of the Princess of Argos and receiving part part of the kingdom as reward. In fact, small doses of hellebor have laxative properties but high doses can be toxic; it is a polyvalent drug that ancient doctors knew they had to carefully manage (they had already observed the 'dose-dependent' effect of the 'pharmacokon').

The Ancient Greek word 'hellebor' origins from the two words 'ellòs' ('fawn') and 'bibróskein' ('eaten'), so it means 'plant eaten by fawns'. In facts, Fawns were known to possibly die from excessive consumption of hellebore. The definition of hellebor is therefore equivalent to the expression 'mortal feeding', thus, even hellebor could represent an example of 'pharmacon'.

While an excessive amount of black bile was the cause of melancholia, the yellow one was supposed to be responsible for pathologic mood elevation ('mania'). Aristoteles (384 to 322 BCE) considered melancholia and mania as 'dysfunctions of the body structure' [19]. Indeed, the first author linking together the two essential, opposite polarity moods was Aretaeus the Cappadocian (81 to 138 AD): ' [...] I think that melancholia is the beginning and part of mania' [20].

Further investigations on mood disorders were performed later by Galenus (129 to circa 200 or 216 AD), who considered melancholia to be due to brain alterations induced by black bile intoxication [21]. Interestingly, Ancient Greeks already observed that many psychic conditions were (also) due to environmental and dietary factors, among others. Indeed, such valuable observations further stimulated 'pharmacological' therapies even during later years when psychic and somatic conditions were still seen as distinct phenomena.

For example, the CYP3A-inducing enzymatic effect of hypericum [22] was obviously unknown during the Middle Ages and for a long time after, however the hypericum plant was extensively used with the name of 'Saint John's Wort' to treat gout, intestinal bleeding and liver diseases and also as a 'psychopharm', to 'ward off evil spirits' considered responsible for depression [23].

Remarkably, recent studies have demonstrated hypericum to have potential antidepressant properties provided by one of its active components, hypericin [23]. Hypericum extracts inhibit norepinephrine, dopamine and serotonin reuptake, increasing the concentration and the number of presynaptic and postsynaptic serotonergic receptors and strengthening γ-aminobutyric acid (GABA)ergic transmission by directly stimulating the GABA receptors [24].

Also, numerous solanaceae family plants such as atropa belladonna, stramonium (thorn apple), hyoscyamus (henbane), and mandrago officinalis (mandrake), were invested with therapeutic and magic properties such as sleep-inducing, analgaesic, antihistaminic and hallucinogenic ones, related to their anticholinergic action exerted both on the CNS and PNS [10].

Mandrake, with its dreadful anthropomorphic roots (there was a 'male' and a 'female' plant), was considered as a magic element and was therefore traded for its supposed aphrodisiacal effects. According to legend, the mandrake grew 'in the shade of the gallows' and 'where the tears of people sentenced to death had fallen' or 'in places frequented by witches and demons'. The legend also reports that if uprooted, the plant uttered a deadly cry able to make people go 'insane' or die. To avoid that, a black dog held on leash was employed during the extraction process "with a magical formula been pronounced in this circumstance". Also this ritual could have been performed only during specific astral conditions [10]. In other words, the "chance to become insane" was associated with magical and obscure causes requiring mysterious rituals to be performed in order to avoid such occurrence.

While Ancient Greeks generally considered psychic and somatic conditions as different manifestations, possibly part of the same illness, in the following centuries physicians often neglected these observations, mainly due to new religious influences.

Yet today it is sometimes reported by the media (and in the worst scenario by few doctors too) and others that 'psychopharms may hurt', and 'psycho'-pharms could be reported to be completely different from 'pharms'. This worrying phenomenon can also happen in cases where the 'pharm' and 'psychopharm' are the exact same molecule.

While most polyvalent actions from the same active principle had already been reported by Ancient Greeks physicians (usually exerted on 'different manifestations of the same illness'), the modern 'psycho' prefix still represents a hard to remove cultural limit in modern psychiatry.

It is curious to observe how psychopharmacology got its prefix just centuries after it was *de facto *introduced. Huge scientific progress has been observed since Ancient Greek times even if sometimes patients and doctors still have difficulty in forgetting the 'psycho' prefix or in overcoming some limitations of the doctor-patient relationship. Indeed, we need more knowledge about these prejudices, and we should carefully 'study the past to divine the future'.

## Conclusion

Ancient Greek mythology and other ancient cultures, independent of age or region, often represent a valuable knowledge source for a better understanding of modern medicine.

Medicine, as the science aimed at treating pain and illness, has always represented a core aspect of human societies. While among most ancient civilizations no difference occurred between medicine, mythology and religion, a core separation seems now almost to have been achieved. However, psychic and somatic conditions can sometimes be 'too separated'.

Surprisingly, most current feelings patients could experience toward doctors (and *vice versa*) and toward their prescriptions, have already been investigated and described in ancient myths. This is probably due to the 'essential nature' of human needs.

Even if today a scientific approach to medicine, psychiatry and drug therapy is the leading one, ancient medicine and related myths represent a useful tool to enhance compliance and clinical outcome, and should therefore be known by a higher number of medical practitioners, especially by psychiatrists.

## Competing interests

The authors declare that they have no competing interests.

## Authors' contributions

MF drafted the manuscript, NC and PF contributed in its revision. All authors read and approved the final manuscript.

## References

[B1] Roccatagliata G (1970). Psychiatric nosology of B. Perdulcis, famous French physician of the XVIIth century. Sist Nerv.

[B2] Morris JG, Donohoe M (2004). The history of hysteria. Pharos Alpha Omega Alpha Honor Med Soc.

[B3] Silbert M (1968). Masked depression and the general practitioner. S Afr Med J.

[B4] Arana GW (2000). An overview of side effects caused by typical antipsychotics. J Clin Psychiatry.

[B5] Weiden PJ (2007). EPS profiles: the atypical antipsychotics are not all the same. J Psychiatr Pract.

[B6] Meyer SE, Chrousos GP, Gold PW (2001). Major depression and the stress system: a life span perspective. Dev Psychopathol.

[B7] Bakish D (2001). New standard of depression treatment: remission and full recovery. J Clin Psychiatry.

[B8] D'Anna G (1996). Dizionario dei miti.

[B9] Graves R (1963). I miti greci.

[B10] Magno PL (1994). Storie di piante medicinali eccellenti. XXX: Ciba-Geigy; Roma, Italia.

[B11] Cosmacini C (1997). L'arte lunga. Storia della medicina dall'antichità ad oggi.

[B12] Yeung A, Chang D, Gresham RL, Nierenberg AA, Fava M (2004). Illness beliefs of depressed Chinese American patients in primary care. J Nerv Ment Dis.

[B13] Campagna JA (2005). The end of religious fatalism: Boston as the venue for the demonstration of ether for the intentional relief of pain. Surgery.

[B14] Fanfani M (1996). L'arte: un potente mezzo d'informazione nell'evoluzione della medicina.

[B15] Moerman DE (2002). Meaning, medicine and the 'placebo effect'.

[B16] Kennedy WP (1961). The nocebo reaction. Med World.

[B17] Scott DJ, Stohler CS, Egnatuk CM, Wang H, Koeppe RA, Zubieta JK (2008). Placebo and nocebo effects are defined by opposite opioid and dopaminergic responses. Arch Gen Psychiatry.

[B18] Ghaemi SN, (Ed) (2002). Polypharmacy in psychiatry.

[B19] Radden J (2000). Review - the nature of melancholy from Aristotle to Kristeva.

[B20] Radden J (2003). Is this dame melancholy? Equating today's depression and past melancholia. Phil Psychiatry Psychol.

[B21] Koelbing HM (1985). Storia della terapia medica Momenti fondamentali.

[B22] Lee JY, Duke RK, Tran VH, Hook JM, Duke CC (2006). Hyperforin and its analogues inhibit CYP3A4 enzyme activity. Phytochemistry.

[B23] Brunello N (1998). Recenti acquisizioni nella farmacoterapia delle sindromi depressive: ruolo dell'iperico.

[B24] Gramowski A, Jügelt K, Stüwe S, Schulze R, McGregor GP, Wartenberg-Demand A, Loock J, Schröder O, Weiss DG (2006). Functional screening of traditional antidepressants with primary cortical neuronal networks grown on multielectrode neurochips. Eur J Neurosci.

